# In Vitro and in Vivo Anti-tumor Activity of miR-221/222 Inhibitors in Multiple Myeloma

**DOI:** 10.18632/oncotarget.820

**Published:** 2013-02-24

**Authors:** Maria Teresa Di Martino, Annamaria Gullà, Maria Eugenia Gallo Cantafio, Marta Lionetti, Emanuela Leone, Nicola Amodio, Pietro Hiram Guzzi, Umberto Foresta, Francesco Conforti, Mario Cannataro, Antonino Neri, Antonio Giordano, Pierosandro Tagliaferri, Pierfrancesco Tassone

**Affiliations:** ^1^ Department of Experimental and Clinical Medicine, Magna Graecia University, Catanzaro, Italy; ^2^ Medical Oncology, Tommaso Campanella Cancer Center, Catanzaro, Italy; ^3^ Department of Medical Sciences University of Milan, Hematology1, IRCCS Policlinico Foundation, Milan, Italy; ^4^ Department of Medical and Surgical Sciences, Laboratory of Bioinformatics Unit; ^5^ Department of Human Pathology and Oncology, University of Siena, Siena, Italy; ^6^ Sbarro Institute for Cancer Research and Molecular Medicine, Center for Biotechnology, College of Science and Technology, Temple University, Philadelphia, PA, USA

**Keywords:** miR-221, miR-222, microRNA, miRNA, multiple myeloma, plasma cell leukemia

## Abstract

A rising body of evidence suggests that silencing microRNAs (miRNAs) with oncogenic potential may represent a successful therapeutic strategy for human cancer. We investigated the therapeutic activity of miR-221/222 inhibitors against human multiple myeloma (MM) cells. Enforced expression of miR-221/222 inhibitors triggered in vitro anti-proliferative effects and up-regulation of canonic miR-221/222 targets, including p27Kip1, PUMA, PTEN and p57Kip2, in MM cells highly expressing miR-221/222. Conversely, transfection of miR-221/222 mimics increased S-phase and down-regulated p27Kip1 protein expression in MM with low basal miR-221/222 levels. The effects of miR-221/222 inhibitors was also evaluated in MM xenografts in SCID/NOD mice. Significant anti-tumor activity was achieved in xenografted mice by the treatment with miR-221/222 inhibitors, together with up-regulation of canonic protein targets in tumors retrieved from animals. These findings provide proof of principle that silencing the miR-221/222 cluster exerts significant therapeutic activity in MM cells with high miR-221/222 level of expression, which mostly occurs in TC2 and TC4 MM groups. These findings suggest that MM genotyping may predict the therapeutic response. All together our results support a framework for clinical development of miR-221/222 inhibitors-based therapeutic strategy in this still incurable disease.

## INTRODUCTION

Multiple myeloma (MM) is a hematologic malignancy characterized by proliferation of neoplastic plasma cells in the bone marrow [1-3]. Despite a considerable improvement of MM patient survival has been recently achieved, the course of this disease remains lethal in most of cases [1, 2, 4, 5]. However, the recent availability of novel research platforms and of a variety of investigational therapeutic options is hopefully leading to improved treatment strategies in MM patients [6-10]. Such advancements are based on a deeper understanding of disease pathobiology [11]. Complex genetic aberrations contribute to the multistep transformation process of plasma cells within the human bone marrow microenvironment (huBMM), which plays an essential role for growth, survival and drug resistance of tumor cells [12-14]. There is now a rising body of evidence that these aberrations may deregulate the microRNA (miRNA) expression in MM cells [15-17], which in turn results into altered protein translation of messenger RNAs. In this complex scenario, the miRNA network is progressively disclosing its key involvement in the MM pathogenesis suggesting potential relevant clinical applications in this important disease [18-22].

MicroRNAs are a class of regulatory non-coding RNAs of 19–25 nucleotides which act by targeting specific messenger RNAs (mRNAs) for degradation or inhibition of translation through base pairing to partially or fully complementary sites [16, 23, 24]. At present, the miRNA network, which includes several hundred sequences, is known to be involved in a variety of normal biological functions [25, 26] as well as in tumorigenic events [27] since, deregulated miRNAs can act either as oncogenes or tumor-suppressors [28-30] and depending on cellular contexts [31]. Epigenetic and/or genetic changes account for miRNA deregulation, as well as different transcription factors may activate the miRNA promoter after it is epigenetically activated [32]. Therefore, miRNAs are emerging as intriguing multi-protein target regulators in the context of signaling networks involved in cancer promotion or repression as well as biomarkers for the disease progression [33].

Among miRNAs significantly deregulated in human cancer, miR-221/222 are of major interest as potential targets for therapeutic applications. miR-221/222 are highly homologous miRNAs encoded in tandem on the X chromosome, whose up-regulation has been recently described in several types of human tumors. miR-221/222 act as oncogenic miRNAs that facilitate cell proliferation via down-regulation of p27Kip1 and/or p57Kip2 [34-36], which negatively regulate cell cycle progression from G1- to S-phase. Several reports suggested a key role of miR-221/222 in tumorigenesis. In fact, it has been recently shown that up-regulation of miR-221/222 expression, confers resistance to TRAIL-induced cell death and enhances proliferation and cell survival of lung and liver cancer cells by targeting PTEN and TIMP3 [37]. Moreover, it has been demonstrated that miR-221/222 regulate radiosensitivity, cell growth and invasion of gastric cells by modulation of PTEN expression [38]. More recently, it has been reported that the treatment with LNA-modified miR-221 inhibitors reduces the growth of liver cancer cells over-expressing miR-221/222 *in vitro* by targeting a DNA damage-inducible transcript 4 (DDIT4), a modulator of the mTOR pathway [39]. In addition, other authors recently showed that miR-221/222 antisense oligonucleotides reduce tumor growth by increasing intra-tumor p27Kip1 protein expression [40]. Taken together, all these findings strongly support the notion that silencing miR-221/222 may represent a highly promising therapeutic option that warrants further investigation in other malignancies.

Since the therapeutic potential of miR-221/222 selective inhibitors has not before investigated in MM, we studied and report here the biological effects induced by miR-221/222 *in vitro* and *in vivo* silencing. Our results support the development of miR-221/222 inhibitors as novel agents for the treatment of MM.

## RESULTS

### Expression of miR-221/222 in MM and PCL patients, and in MM cell lines

**Figure 1:**
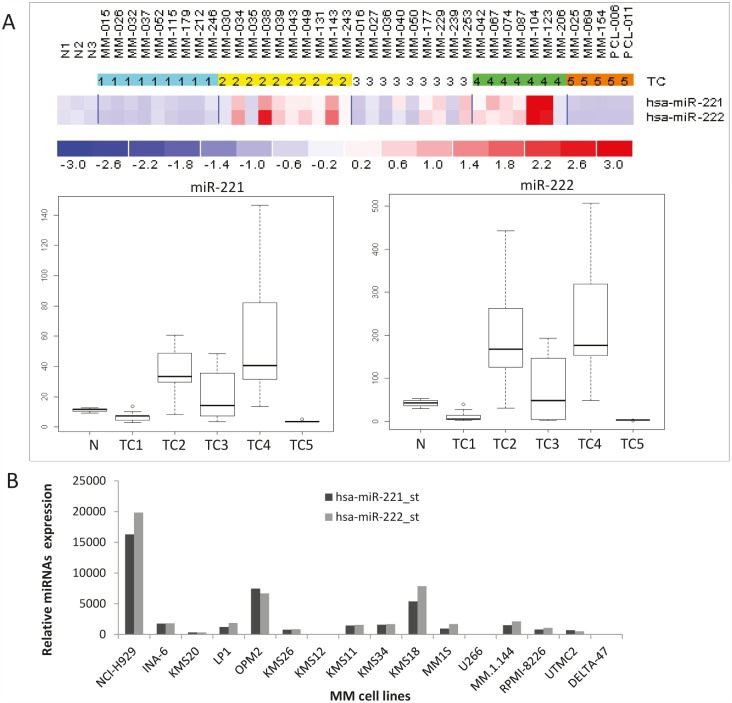
miR-221 and miR-222 expression in primary CD138+ normal plasma cells, primary MM and PCL cells and established MM cell lines A) Differential expression of miR-221 and miR-222 in immunoselected CD138+ cells from 3 healthy donors, 38 MM and 2 PCL, by microarray analysis. Expression values were normalized by aroma.light-package for Bioconductor. MM were TC classified according to the presence of the recurrent IGH chromosomal translocations and cyclins D expression as previously described (30). miR-221 and miR-222 are reported as raw expression values. Statistical significance was assessed by SAM multi-class analysis, (q-value=0). N(1-3): CD138+ cells from normal healthy donors. MM and PCL were numbered referring to individual patients in the original data set. B) Differential expression of miR-221 and miR-222 in 16 MM cell lines by Affymetrix GeneChip® miRNA 1.0 Array. Histogram bars indicate miR-221 or miR-222 expression values normalized by miRNA QC Tool (Affymetrix).

Figure 1A shows the heatmap of miR-221/222 expression in a panel of CD138+ cells from 38 MM patients, 2 PCL patients and plasma cells from 3 healthy donors previously investigated by microarray analysis [15]. Among different TC (Translocation/Cyclin) classified MM samples, we found significantly higher miR-221/222 expression in TC2, TC4 and in a subgroup of TC3 MM, as assessed by SAM multi-class analysis, (q-value=0) (Fig. 1A). Moreover, we evaluated by microarray miRNA profiling the miR-221/222 expression in 16 MM cell lines (Fig. 1B). Among these cells, we selected the U266 t(1;11) and RPMI-8226 t(1;14) cells which express very low levels of miR-221/222 to evaluate the growth promoting role of miR-221/222 mimics. Conversely, we selected OPM2 and NCI-H929 cells, both t(4;14), which respectively express moderate and high levels of miR-221/222 to explore the anti-tumor activity of miR-221 and/or miR-222 inhibitors.

### *In vitro* enforced expression of synthetic miR-221/222 mimics in MM cells

**Figure 2:**
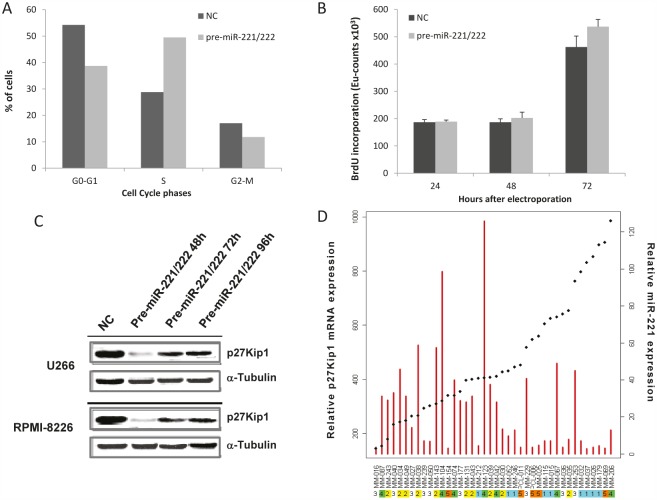
Biological effects induced by transient expression of miR-221/222 in MM cell lines A) Cell cycle perturbation in U266 cells induced by transient pre-miR221/222 enforced expression. At least 20,000 events for each point were analyzed in 3 independent experiments. Results are representative of one out of 3 experiments 72 hours after transfection. B) Effects induced on BrdU uptake of RPMI-8226 cells by transfection with pre-miR-221/222 mimics or scrambled sequences. Averaged values ±SD from 3 independent experiments are plotted. C) p27Kip1 protein expression 48-72 and 96 hours after transfection of U266 and RPMI-8226 cells with pre-miR-221/222 mimics or scrambled controls (NC) was assessed by Western blotting assay. D) Inverse correlation between p27Kip1 mRNA and miR-221 levels in a dataset of 40 (38 MM + 2 PCL) patients is shown. The red bars represent mature miR-221 expression (vertical axis on right side) and the spots represent target gene (p27Kip1 mRNA) expression (vertical axis on left side). Horizontal axis: patient samples ordered according to increasing p27Kip1 expression.

We first investigated the growth promoting activity of miR-221/222 by enforced expression of their synthetic mimics in MM cells. To this end, we transfected U266 and RPMI-8226 cells, that constitutively express very low levels of the miRNA-cluster, with miR-221/222 mimics or scrambled oligonucleotides. In transfected U266 cells, we indeed observed an increase in the percentage of cells in S-phase, which become evident after 48h, peaked at 72h and decreased at 96h (Fig. 2A). The increase of S-phase was also detected by Bromodeoxyuridine (BrdU) incorporation in RPMI-8226 cells that reached significant levels 72 hours after transfection. Since miR-221/222 negatively regulates p27Kip1 expression in different cell types [34, 40, 41], we evaluated if this effect also occurred in transfected U266 cells. By Western blotting analysis of whole cell lysate 48h after transfection, we found >90% reduction of p27Kip1 as compared to controls, which begins to raise towards control levels at 72h and 96h time points (Fig. 2C, top panel). Targeting of p27Kip1 protein by miR-221/222 was also evaluated in RPMI-8226 cells, expressing moderate levels of these miRNAs. Again, enforced increase of miR-221/222 resulted in a marked reduction of p27Kip1 protein (Fig. 2C, bottom panel).

We then analyzed the correlation between miR-221/222 and p27Kip1 mRNA expression, as measured by microarray analysis, in a same dataset of MM patients: we found a significant inverse correlation between miR-221/222 and p27Kip1 mRNA (Pearson product-moment correlation, *p*<0.05) (Fig 2D).

### *In vitro* enforced expression of miR-221/222 inhibitors in MM cells

**Figure 3:**
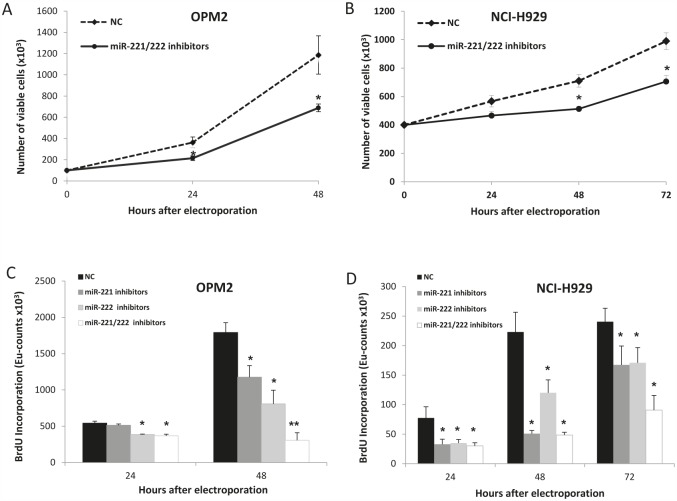
*In vitro* anti-proliferative activity of miR-221 and miR-222 inhibitors on MM cell lines A-B) Cell growth analysis of OPM2 (A) and NCI-H929 (B) cells transfected with miR-221/222 inhibitors or scrambled oligonucleotides (NC). Analysis was performed by trypan blue exclusion assay. Averaged values of 3 independent experiments are plotted in each frame including +SD. Significant P values (P<0.01) are indicated by stars. C-D) BrdU incorporation after transfection of synthetic miR-221 and/or miR-222 inhibitors or NC in OPM2 (C) and NCI-H929 (D) cells. Averaged values of 3 independent experiments are plotted in each frame including +SD. (*) P<0.05, (**) P<0.01.

**Figure 4:**
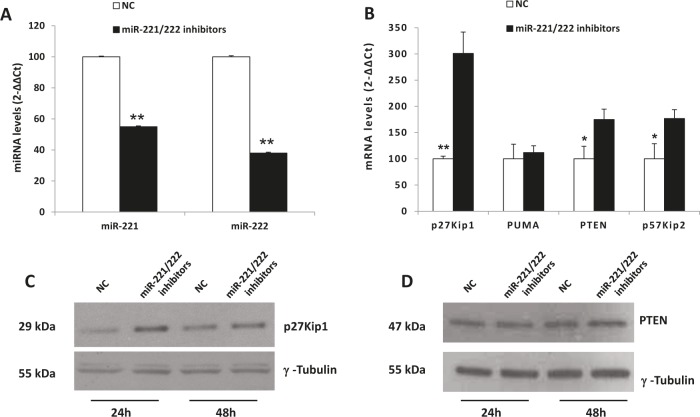
Molecular effects induced by miR-221/222 inhibitors in MM cells A) miR-221 and miR-222 q-RT-PCR 24 hours after transfection with miR-221/222 inhibitors and NC in OPM2 cells. The results are shown as miRNA expression level after normalization with RNU44 and ΔΔCt calculations. Data represent the average of 3 independent experiments +SD. B) q-RT-PCR of p27Kip1, PUMA, PTEN and p57Kip2 mRNA expression 24 hours after transfection with miR-221/222 inhibitors or NC in OPM2 cells. The results are shown as average mRNA expression after normalization with GAPDH and ΔΔCt calculations. Data represent the average of 3 independent experiments +SD. (*) P<0.05, (**) P<0.01. C-D) Western blot analysis of p27Kip1 and PTEN in OPM2 cells 24 and 48 hours after transfection with miR-221/222 inhibitors and NC. γ-tubulin was used as protein loading control.

To investigate the anti-tumor potential of miR-221/222 inhibitors, we transfected OPM2 and NCI-H929 cells, which respectively express moderate-high to high levels of miR-221/222, with miR-221/222 inhibitors. As assessed by trypan blue exclusion assay (Fig. 3A and B) and BrdU cell uptake (Fig. 3C and D), we found significant anti-proliferative activity induced by miR-221/222 inhibitors in both cell lines. To evaluate if this effect correlated with knocking down of miR-221/222, we firstly evaluated the expression of miR-221 and miR-222 by quantitative real time PCR (q-RT-PCR) 24 hours from cell electroporation. We detected an about 50% reduction of both miRNAs in OPM2 MM cells (Fig. 4A), which occurred together with a significant up-regulation of p27Kip1 mRNA (Fig. 4B) and >3-fold increase of p27Kip1 protein expression (Fig. 4C). Moreover, we evaluated the effects induced on others validated miR-221/222 canonic targets, including PUMA, PTEN, and p57Kip2 mRNAs. As shown in Figure 4B, we observed a weak modulation of PUMA mRNA, while PTEN and p57Kip2 mRNAs were >2-fold increased. At protein level, we found increase of p27Kip1 and PTEN proteins 24 and 48 hours after transfection with miR-221/222 inhibitors. We did not detect any modulation of p57Kip2 protein (data not shown), suggesting that this protein is under a different mechanism of expression control in this cell system. All together these results demonstrate that synthetic miR-221/222 inhibitors exert significant anti-tumor effects *in vitro* and this activity involves modulation of several validated targets of miR-221/222.

### Effects of miR-221/222 inhibition on the whole cell transcriptome

**Figure 5:**
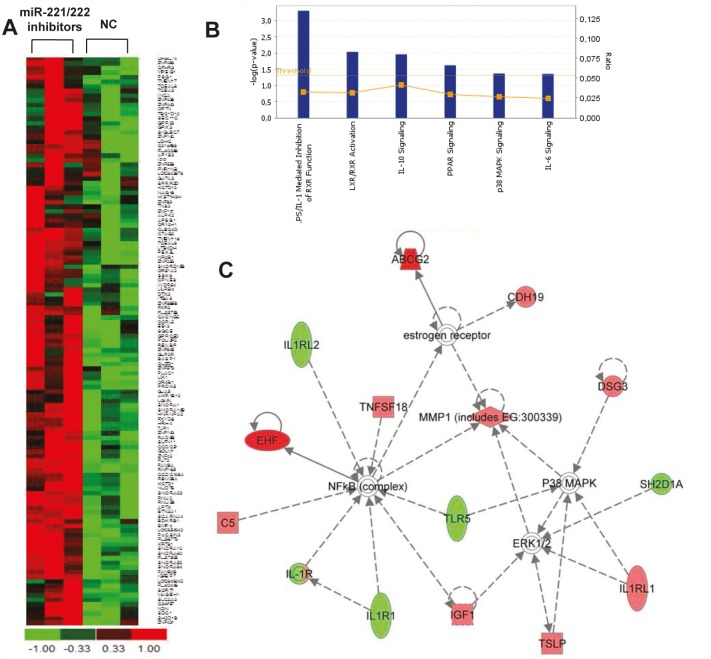
Whole Gene Expression Profiles of MM cells after miR-221/222 knockdown A) Hierarchical clustering of differentially expressed genes between miR-221/222 inhibitors or NC treated OPM2 cells by Gene 1.0 ST array chip (Affymetrix) and DChip software. Genes that underwent a 0.5-fold change as compared to control, were selected and clustered. Assays performed in triplicate are shown. B) Pathways modulated by miR-221/222 inhibitors in OPM2 MM cells are shown. Analysis was performed by Ingenuity Pathway Analysis on genes significantly modulated (FC>0.5). The bar graphs show pathways most modulated by miR-221/222 inhibitors as compared to control, based on statistical significance (P-value and ratio). The yellow line indicates the threshold of significance. C) Functional network scenario with principal nodes in the most perturbed pathways are showed. Up- and down-regulated genes are indicated in red and green, respectively. Continuous and discontinuous lines represent direct and indirect functional and physical interactions between genes as previously reported.

We next investigated the effect induced by miR-221/222 inhibition on OPM2 cells at trascriptome level by performing gene expression analysis. Hierarchical clustering was performed based on differential gene expression after 24 hours of miR-221/222 silencing as compared to the control (Fig. 5A). The figure 5A shows the up-regulation of about one hundred (112) genes that also exhibit similar expression patterns in replica experiments.

To analyze higher-order influences on biological networks regulated by miR-221/222 inhibitors, we focused on gene list obtained after fold change analysis and results underwent Ingenuity Pathway Analysis (IPA). We included into the analysis genes that showed a FC ratio bigger than 0,5. We found a significant modulation of pathways involved in relevant biological functions as cell death, cell cycle, growth and proliferation as well as cell-to-cell signaling and interaction (p-value<0.05). In particular, we found perturbation of signal transduction pathways (i.e. LPS/IL-1 mediated inhibition of RXR function P-value 5.02E-04, LXR/RXR activation P-value 9.28E-03, and IL-10 signaling P-value 1.11E-02, Fig. 5B). These findings suggest that RXR mediated pathways may have an important role in the anti-proliferative effect on MM cells as evidenced by *in vitro* experiments. Moreover, we looked at the mostly perturbed networks. Among these, the network illustrated in Figure 5C appeared of interest with the central nodes represented by NFkB and P38MAPK-ERK1/2 molecules which are connected to up-regulated (i.e. EHF, IGF1, MMP1, IL1RL1) or down-regulated (i.e. SH2D1A, IL1R2, TLR5) genes. These findings led to the identification of pathways involved in anti-tumor mechanisms of miR-221/222 inhibitors suggesting novel opportunities for the rational design of combinatory strategies with signaling inhibitors.

### *In vivo* anti-tumor activity of miRNA-221/222 against MM xenografts

**Figure 6:**
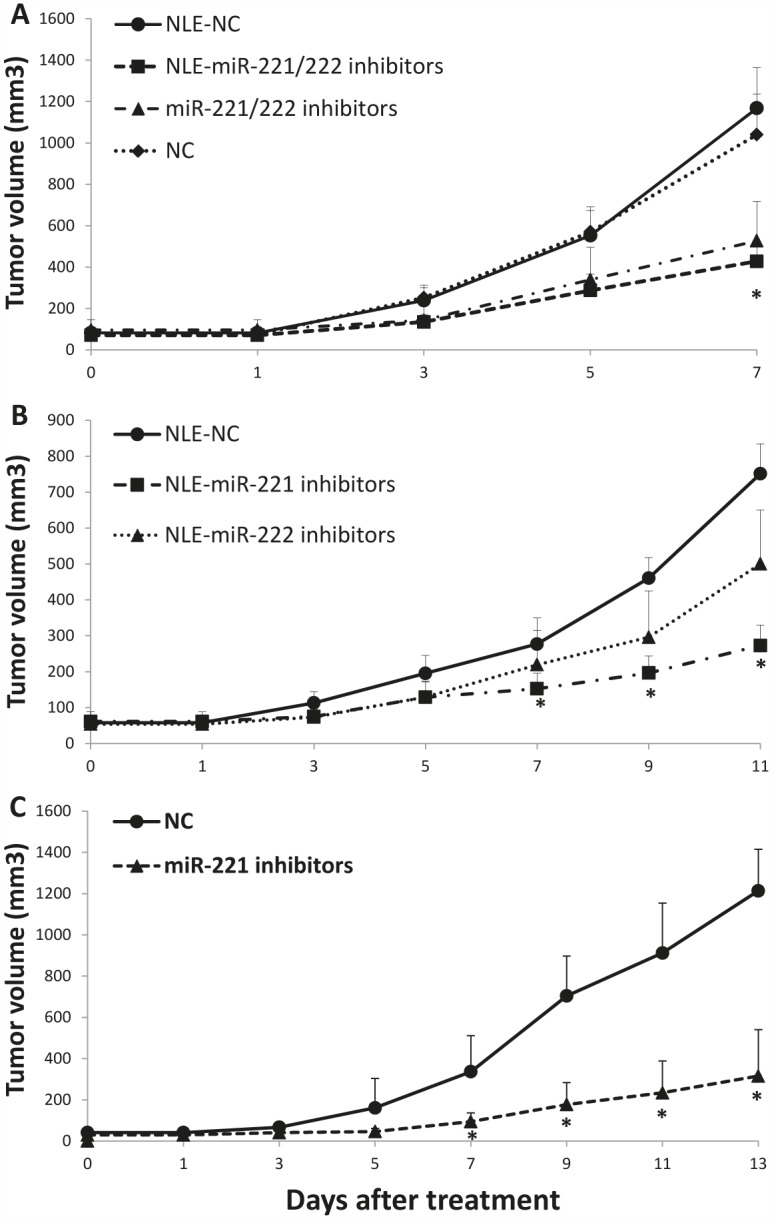
*In vivo* anti-tumor activity of miR-221/222 inhibitors in MM xenografted SCID/NOD mice A) Effects of formulated or unformulated miR-221/222 inhibitors. Subcutaneous OPM2 xenografts were treated every 2 days with 20 μg of formulated(NLE)-miR-221/222 inhibitors or unformulated-miR221/222 inhibitors. As control 2 separate groups of tumor-bearing animals were injected with formulated(NLE)-NC or unformulated-NC. B) Effects of treatments with formulated miR-221 or miR-222 individual inhibitors. Subcutaneous OPM2 xenografts were repeatedly treated every 2 days, with 20 μg of NLE-miR-221 inhibitors, or NLE-miR-222 inhibitors, or NLE-NC. C) Effects of unformulated-miR-221 inhibitors or NC. Subcutaneous OPM2 xenografts were repeatedly treated every 2 days with 20 μg of unformulated-miR-221 inhibitors, or unformulated-NC. The tumor volumes averages of each group +SD are reported. In each experiment P values were calculated for miRNA inhibitors *versus* scrambled oligonucleotides (NC) and significant P values (*P*<0.05) are indicated by stars.

**Figure 7:**
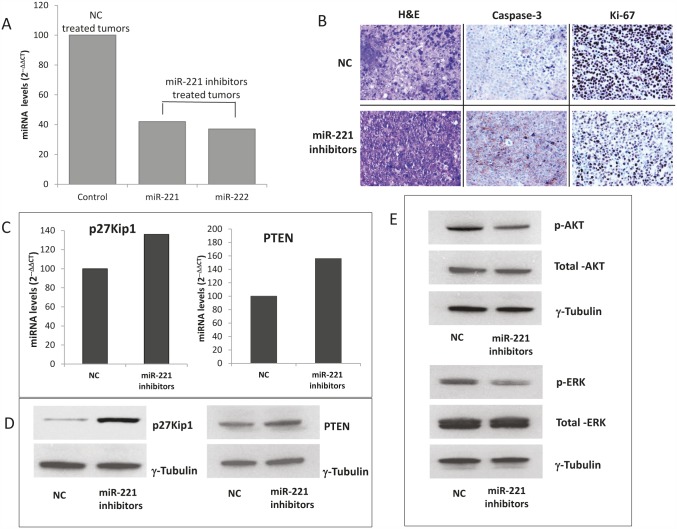
miR-221 activity and targets silencing in retrieved MM xenografted tumors A) q-RT-PCR of miR-221 and miR-222 in retrieved tumors treated with unformulated-miR-221 inhibitors or unformulated-NC. The results are shown as averaged miRNA expression after normalization with RNU44 and ΔΔCt calculations as compared to control (miRNA expression in NC treated animals). B) H&E (20-fold magnification) and immunohistochemistry (40-fold magnification) staining of xenografted tumors retrieved from treated animals. C) q-RT-PCR of p27Kip1 and PTEN mRNA levels in treated tumors retrieved from mice. Raw Ct values were normalized to GAPDH housekeeping mRNA and expressed as ΔΔCt values calculated using the comparative cross threshold method. D) Western blot analysis of p27Kip1 and PTEN protein in retrieved tumors from mice treated with miR-221 inhibitors or NC. D-E) Western blot analysis of total AKT and p-AKT (D) and total ERK and p-ERK (E) in retrieved tumors from mice treated with miR-221 inhibitors or NC. Representative experiments are shown in figure. γ-tubulin was the protein loading control.

In the light of translation of our findings in a therapeutic model, we investigated the effect of miR-221 and/or miR-222 inhibitors against MM xenografts in CB-17 severe combined immunodeficient non-obese diabetic (SCID/NOD) mice. When OPM2 MM tumors became palpable, animals were randomized and treated with miR-221 and/or miR-222 inhibitors or controls. miRNAs inhibitors were both administered as lipidic-formulated with neutral lipid emulsion (NLE) particles that have been demonstrated to successfully deliver oligonucleotides *in vivo* [19, 21, 42, 43] or as unformulated agents. In a first series of experiments, by both delivery strategies, we found a significant (*P<0.05*) anti-tumor activity of miR-221/222 inhibitors against MM xenografts (Fig. 6A). We then compared the activity of single inhibitors and we observed higher anti-tumor activity of miR-221 inhibitors as compared to miR-222 inhibitors (Fig. 6B). On these findings, we further studied the activity of miR-221 inhibitors even as unformulated agents confirming their relevant anti-MM potential in xenografted mice (Fig. 6C). Importantly, by q-RT-PCR analysis of tumor tissues retrieved from animals treated with miR-221 inhibitors, we found a >60% knocking down of both miR-221 and miR-222 expression (Fig. 7A), confirming the *in vivo* interaction between miR-221 and miR-222. Moreover, by histologic and immunohistochemical analysis or retrieved tumors, we observed large areas of necrosis with abundant nuclear debris (“dustlike” nuclear fragments) in xenografts treated with miR-221 inhibitors. In addition, we found that miR-221 triggered apoptosis in MM xenografts *in vivo* with increased cleaved caspase-3 and also reduced Ki-67 expression (Fig. 7B). Finally, by q-RT-PCR and Western blotting, we demonstrated up-regulation of p27Kip1 and PTEN validated miR-221/222 targets at mRNA (Fig. 7C) and protein (Fig. 7D) level, respectively. Notably, inhibition of miR-221/222 significantly reduced the phosphorylation of AKT, a down-stream target of PTEN and key mediator of tumor cell survival (Fig. 7E), suggesting that the *in vivo* anti-MM activity of miR-221 inhibitors is related to PTEN up-regulation and impairment of AKT activation within tumors. Interestingly, the inhibition of miR-221/222 *in vivo* considerably also reduced the p-ERK1/2 levels, while total ERK1/2 was unaffected. Taken together, these results demonstrate the therapeutic potential of miR-221 inhibitors in validated preclinical *in vivo* models and provide important molecular insights.

## DISCUSSION

In this report, we show that miR-221/222 is significantly up-regulated in a subset of MM patients that by translocation and cyclin (TC) molecular classification mostly include TC2, TC4 and a subpopulation of patients within TC3 group, overall accounting for >50% of all MM. Most importantly, we demonstrate that silencing these miRNAs by specific inhibitors in MM cells bearing t(4;14) results in a powerful anti-tumor activity *in vitro* and in clinically relevant murine models of human MM. To our knowledge, this is the first experimental evidence of anti-tumor activity of miR-221/222 inhibitors against MM cells.

An additional important achievement of our work is the successful delivery of both lipid-based formulated [43] or unformulated miR-221/222 inhibitors against MM xenografts. This point is relevant since the optimal bioavailability of unformulated miR-221/222 will support the design of therapeutic strategies without the need of delivery systems. This high stability of single-strand oligonucleotides miRNA inhibitors may be due to chemical stabilization of RNA sequences. This point is of obvious relevance in the translation of miRNA inhibitors for clinical studies. Importantly, the down-regulation of canonic miR-221 targets in tumors excised from treated animals confirms the successful tumor-uptake of miRNA inhibitors at concentrations sufficient to produce up-regulation of target mRNAs and proteins that in turn translates in anti-tumor effects.

Over-expression of miR-221/222 has been observed in a number of advanced solid tumors indicating that miR-221/222 could be potential therapeutic targets also for hematopoietic malignancies. The molecular mechanisms of anti-MM activity of miR-221/222 inhibitors are presently under investigation. An important target of miR-221/222 is the CDK inhibitors p27Kip1, one of the most important cell cycle regulator, which has relevant impact on the proliferation and cell cycle control in a variety of human malignancies, including prostate carcinoma [40], glioblastomas [35], thyroid carcinomas [44], breast cancer [45], hepatocellular carcinoma [46], and lung cancer [47]. In our experimental setting, MM cells, 24 hours after transfection with miR-221/222 mimics with low/moderate basal miR-221/222 expression, significantly increase the S-phase that conversely decreases after miR-221/222 inhibitor treatment. A further important oncosuppressor gene target identified is the p57Kip2 that is reported as miR-221/222 direct target in the liver, whose suppression is involved, with an oncogenic function, in hepato-carcinogenesis [48]. In our *in vitro* experiments, we observed up-regulation of both CDK inhibitors, p27Kip1 and p57Kip2, suggesting that these molecules are relevant players in cell cycle regulation and proliferation of MM cells. Among the most important miR-221/222 gene targets, the tumor suppressor PTEN was observed to negatively regulate glioma cell migration [49]. PTEN functions as tumor suppressor by negatively regulation of AKT/PKB signaling pathway. The increase of PTEN mRNA level detected after miR-221/222 inhibitor transfection leads us to speculate that the major anti-proliferative effect on MM cells is mediated by cell cycle regulation. In fact, we were not able to detect apoptosis *in vitro*. This evidence together with the minor changes of BBC3/PUMA mRNA, a p53 modulator of apoptosis and validated target of miR-221/222, provides additional support to our hypothesis. The *in vivo* immunohistochemical analysis of apoptosis in retrieved miR-221 inhibitor-treated tumors indicates however activation of caspase-3. This observation suggests that other players, for istance perturbation of angiogenesis, trigger the apoptotic cascade *in vivo*.

Genome wide mRNA expression after miR-221/222 knockdown identified modulation of canonic pathways involved in cell proliferation signals and activation of immune response. By Ingenuity Pathway Analysis, we identified pathways involved in tumor growth, oncogenesis, invasiveness and progression. Among these, the RXR modulation seemed of particular interest since it is ligand-dependent nuclear receptor (NR) that forms a complex with other ligand-dependent NRs and transcriptional receptors and it is directly involved in signal transduction, cell proliferation, morphology and apoptosis [50]. Interestingly, other pathways that positively interact with RXR, such as p38MAPK signaling, IL-6 and IL-10 signaling were also affected. Other genes and pathways involved in tumor progression were also significantly modulated. Intriguingly, EHF transcription factor and MMP1 were among the most up-regulated genes. EHF is a member of ETS homologus transcription factor and it was recently reported that re-expression of ESE3/EHF inhibited tumorigenic potential of prostate cancer [51], while MMP1 a cell surface metallopeptidase is a miR-221/222 and miR-1928 families target [52]. All together, these data strengthen the molecular framework for the development of rationally designed therapeutic combinations.

In conclusion, the anti-tumor activity of miR-221 inhibitors in clinically relevant mouse models, together with the specific modulation of canonic miR-221/222 targets, provide a robust framework for clinical development of synthetic miR-221 inhibitors as novel therapeutics in MM.

## METHODS

### Cells

Human myeloma cell lines U266 were cultured in Iscove's modified Dulbecco's medium (IMDM) while OPM2, NCI-H929, and rpmi-8226 were grown in RPMI-1640 medium (Gibco®, Life Technologies, Carlsbad, CA) as previously described. Briefly, cultures were supplemented with 10% fetal bovine serum (Lonza Group Ltd., Switzerland), 100 U/ml penicillin, and 100 mg/ml streptomycin (Gibco®, Life Technologies) at 37°C in a 5% CO_2_ atmosphere. Primary CD138+ patient MM and PCL cells were obtained by Ficoll gradient separation followed by positive selection from patient BM aspirates, using CD138 MicroBeads antibody (MACS, Milteny Biotec) [53]. TC classification according to the presence of the recurrent IGH chromosomal translocations and cyclins D expression [54].

### *In vitro* transfection of MM cells by synthetic miR-221/222 mimics or inhibitors

MM cell transfection has been performed, by the Neon® Transfection System (Life Technologies) at 100 nM of pre-miRNAs or miRNAs inhibitors concentrations (Life Technologies). Non-targeting pre-miRNAs or miRNAs inhibitors (negative controls, NC) were used at the same concentration as previously described [19]. Cells were collected and processed for q-RT-PCR (TaqMan miRNA assays, Applied Biosystems), immunoblotting (WB), proliferation assay (Trypan blue exclusion assay, BrdU incorporation, caspase activation) and cell cycle analysis by flow cytometry at different time points after transfection (24, 48, 72 and 96h).

### Cell proliferation assay

For cell proliferation analysis, 1.5x10^5^ MM cells were seeded in 24 well plates, electroporated with synthetic miR-221 and/or miR-222 or with miR-inhibitors or NC, and then harvested and counted at 24-hour intervals using a Trypan Blue viable cell-excluding assay. Each assay was performed in triplicate samples. BrdU uptake assay was performed by measuring the incorporation of BrdU into newly synthesized DNA strands using the DELFIA cell proliferation kit (Perkin-Elmer, Waltham, Massachussets) according to the manufacturer's instructions. Briefly, after transfection with miRs, or miRs-inhibitors or NC, 1x10^4^ cells were plated in 96 well. 10 μM BrdU was added at 24 hours intervals and BrdU-incorporation was measured by time-resolved fluorescence of a europium-chelate on a Wallac Victor^2^ multilabel counter (Perkin-Elmer, Waltham, Massachusetts). All assays were repeated at least twice.

### Quantitative real-time amplification of miRNAs and mRNAs

For q-RT-PCR, 15 ng of total RNA, prepared with the TRI*z*ol® Reagent (Invitrogen) according to manufacturer's instructions, underwent reverse transcription by the Taq-Man® MicroRNA RT Kit or High Capacity cDNA Reverse Transcription Kit (Life Technologies) and specific miRNA or mRNA primers, according to the manufacturer's instructions. Real-time PCR was performed using TaqMan® MicroRNA Assays together with the TaqMan®Fast Universal PCR Master Mix on an ViiA7 System (Life Technologies). The miRNA or mRNA expression was quantified using the 2^−ΔΔCt^ method (Applied Biosystems User Bulletin No. 2), and expressed as the relative quantity of target miRNA or mRNA normalized to the RNU44 (assay ID 001094) or GAPDH (assay ID Hs03929097_g1) housekeeping gene, respectively. Comparative real-time polymerase chain reaction was performed in triplicate, including no-template controls. Relative expression was calculated using the comparative cross threshold (Ct) method [55].

### Western blotting analysis

SDS-PAGE and WB were performed according to standard protocols. Briefly, cells were lysed in lysis buffer containing 15mM Tris/HCl pH 7.5, 120mM NaCl, 25mM KCl, 1mM EDTA, 0.5% Triton 100, Halt Protease Inhibitor Single-Use cocktail (100X, Thermo Scientific). Whole cells lysates (50 μg per line) from transfected cell lines were separated using 4-12% Novex Bis-Tris SDS-acrylamide gels (Invitrogen), electro-transferred on Nitrocellulose membranes (Bio-Rad), and immunoblotted with the following antibodies: p27Kip1 (SX53G8.5) mouse mAb (Cell Signaling), PTEN (A2B1) rabbit mAb (Santa Cruz), Phospho-AKT (Ser473) rabbit mAb (Cell Signaling), AKT (pan, 11E7) rabbit mAB (Cell Signaling), Phospho-p44/42 MAPK (Erk1/2) (Thr202/Tyr204) rabbit mAb (Cell Signaling), p44/42 MAPK (Erk1/2) (Thr202/Tyr204) rabbit mAb (Cell Signaling), γ-Tubulin antibody (C-20) goat polyclonal (Santa Cruz). Membranes were washed 3 times in PBS-Tween, and then incubated with a secondary antiboby conjugated with horseradish peroxidase in 0.5% milk for 2 hours at room temperature. Chemiluminescence was detected using Pierce ECL Western Blotting Substrate (ID 32109, Pierce). Signal intensity was quantified by Quantity One Analyzing System (Bio-Rad).

### Cell cycle phase distribution analysis

Electroporated MM cells, were plated in 6-well plates (0.5 × 10^6^ cells/ml) and cultured for 24, 48, 72, and 96 hours. At each time, cells were collected, washed twice in ice/cold 1x PBS and fixed by incubation in 70% ice/cold ethanol at -20 °C o.n. Before cytofluorimetric analysis, 5x10^5^ cells were washed twice in 1x PBS and stained in 10 mg/ml propidium iodide, 100 mg/ml RNase, 0.05% Nonidet P-40 for 5 hours at room temperature in the dark. Cell cycle profiles were determined using MODFIT software (Verity Software House, Topshem, ME, USA) on a FACScan flow cytometer (Becton Dickinson, San Jose, CA, USA).

### Gene-expression profiling

Gene expression profiles were obtained from OPM2 cells after transfection with miR-221/222 inhibitors or NC in 3 parallel experiments. 24 hours after transfection cells were collected and used for total RNA (tRNA) extraction by Trizol lysis buffer and column purification with RNeasy kit (Qiagen, Hilden, Germany). A total of 300 ng RNA were used as starting material for preparing the hybridization target by using the Ambion® WT Expression Kit (Ambion, Life Techologies). The integrity, quality and quantity of tRNA were assessed by the Agilent Bioanalyzer 2100 (Agilent Technologies, Santa Clara, CA) and NanoDrop 1000 Spectrophotometer (Thermo Scientific, Wilmington, DE). The amplification of cRNA, the clean up and the fragmentation were performed according to the Affymetrix's procedures. Microarray data was generated by Human GeneChip 1.0 ST (Affymetrix Inc., Santa Clara, Ca) containing 764,885 distinct probes that interrogates 28,869 well-annotated genes. Arrays were scanned with an Affymetrix GeneChip Scanner 3000.

Raw data produced by the Affymetrix Platform (i.e. CEL files) were first processed using Affymetrix Expression Console (EC). Pre-processing phase was performed according to Affymetrix guidelines and micro-CS software. Raw data were normalized using probe logarithmic intensity error (PLIER) algorithm coupled to quantile normalization [56]. Annotation of data was also performed using Affymetrix Provided Libraries and EC version 1.1. Differential expression was assessed using a linear model method. *P*-values were adjusted for multiple testing using the Benjamini and Hochberg method. Tests were considered to be significant for adjusted *P*<0.05. Clustering and fold change (FC) analysis were done using the dChip software [57] comparing relative gene expression of scrambled-oligonucleotide *versus* miR-221/222 inhibitors transfected cells. For each pair of compared samples we calculated FC as follows: FC= log_2_(miR-221/222 inhibitors *versus* NC). The genes lists filtered for fold change >0.5 were applied to Ingenuity Pathway Analysis (IPA^®^) software to reveal biological pathways modulated by miR-221/222 silencing (Ingenuity System, Redwood city, CA).

### Animals and in vivo models of human MM

Female SCID/NOD mice (6- to 8-weeks old; Harlan Laboratories, Inc., Indianapolis) were housed and monitored in our Animal Research Facility. All experimental procedures and protocols have been approved by our University Hospital Institutional Ethical Committee and conducted according to protocols approved by the National Directorate of Veterinary Services (Italy). For our study, we used MM xenografts in SCID/NOD mice [58, 59]. For this model, mice were subcutaneously inoculated in the interscapular area with 1x10^6^ OPM2 cells in 100 μL RPMI-1640 medium. The animal treatment was initiated after the detection of palpable tumors, approximately 2 weeks following MM cells injection with 1mg/kg per mouse of miR-221 inhibitor or miR-222 inhibitor, or both miR-221/222 inhibitors, or NC as control (mirVana custom inhibitor, Life Technologies). The tumor sizes were assessed as previously described. Administration of miRNA inhibitors was performed as unformulated agents or formulated by the use of the NLE (MaxSuppressor *in vivo* RNA Lancer II, BIOO Scientific, Austin, TX) according to the manufacturer's instructions. Treatments were performed by intra-tumor (i.t.) injection every two days for a total of seven injections. Tumors were then collected and placed in either 10% formalin for histology or in RNA*later*® for RNA isolation or stored at -80°C for protein analysis.

### Histology and immunohistochemistry

At the end of observation animals were sacrificed and tumors were retrieved for further analysis. Tumors were immediately immersed in 4% buffered formaldehyde and after 24 h, washed, dehydrated, and finally embedded in paraffin. Staining with ematoxylin-eosin was performed using 4 μm tumors section which were mounted on poly-lysine slides and stained with H&E. Observation were done by light microscopy analysis using an optical microscope Nikon i55 (Nikon Corporation, Tokyo, Japan). For immunohistochemistry staining, tumor slices 2 μm thick were deparaffinized and pre-treated with the Epitope Retrieval Solution 2 (EDTA-buffer pH 8.8) at 98°C for 20 min. After washing steps, peroxidase blocking was carried out for 10 min using the Bond Polymer. All procedures were performed using the Bond Max Automated Immunohistochemistry (Menarini). Tumors were again washed and then incubated with the primary antibody directed against Ki-67 (Dako, clone MIB-1; 1:150) or caspase-3 (Novocastra, clone JHM62; 1:500). Subsequently, tissues were incubated with polymer for 10 min and developed with DAB-Chromogen for 10 min. Slides were counterstained with hematoxylin.

### Statistical analysis

All *in vitro* experiments were repeated at least 3 times and performed in triplicate; a representative experiment was showed in figures. Statistical significances of differences were determined using Student's *t* test, with minimal level of significance specified as *P* < 0.05. Statistical significance of the *in vivo* growth inhibition observed in miRNAs inhibitor-treated mice compared with control group was determined using Student's *t* test. The minimal level of significance was specified as *P* <0.05. All statistical analyses were determined using GraphPad software (www.graphpad.com). Graphs were obtained using Microsoft Office Excel tool.
